# A multi-dimensional view of context-dependent G protein-coupled receptor function

**DOI:** 10.1042/BST20210650

**Published:** 2023-01-23

**Authors:** Maria Marti-Solano

**Affiliations:** Department of Pharmacology, University of Cambridge, Cambridge CB2 1PD, U.K.

**Keywords:** biological networks, cellular localization, drug discovery and design, G-protein-coupled receptors, signalling, systems biology

## Abstract

G protein-coupled receptor (GPCR) family members can sense an extraordinary variety of biomolecules to activate intracellular signalling cascades that modulate key aspects of cell physiology. Apart from their crucial role in maintaining cell homeostasis, these critical sensory and modulatory properties have made GPCRs the most successful drug target class to date. However, establishing direct links between receptor activation of specific intracellular partners and individual physiological outcomes is still an ongoing challenge. By studying this receptor signalling complexity at increasing resolution through the development of novel biosensors and high-throughput techniques, a growing number of studies are revealing how receptor function can be diversified in a spatial, temporal or cell-specific manner. This mini-review will introduce recent examples of this context-dependent receptor signalling and discuss how it can impact our understanding of receptor function in health and disease, and contribute to the search of more selective, efficacious and safer GPCR drug candidates.

## Introduction

GPCR signalling constitutes a central mechanism allowing cells to sense and adapt to changes in their environment. This signalling can originate from receptors responding to a wide variety of cues including light, odours, ions, small molecules, or peptidic hormones. The key role of GPCRs as privileged entry points for the modulation of cell function has also made them the most successful drug target class in the clinic [1–3]. However, although the fundamental role of GPCR signalling in the regulation of cell physiology has been pharmacologically exploited for decades, researchers are still characterising the complex intracellular signalling processes that are elicited upon receptor activation.

Systematic analyses of intracellular coupling across receptors have revealed how, upon activation, single GPCRs often engage with multiple intracellular signal transducers to regulate cell responses [4]. Dissecting which receptor signalling pathways contribute to specific cell responses can dramatically impact our understanding of GPCR biology by: (i) clarifying how changes in signalling pathway composition in different physiological and pathological conditions can impact cell function; (ii) explaining existing variation in therapeutic responses between drugs displaying differential signalling patterns upon binding to the same receptor; and (iii) guiding the rational selection of biased agonists capable of selectively modifying disease-related pathways [5]. Therefore, determining how the activation of different receptor partners contributes to specific cell responses has been a key aim of the GPCR research community [6]. These efforts have been boosted by the development of multiple biosensors [7] and high-throughput assays [8,9] that allow analysing the GPCR signal transduction process with unprecedented detail. Remarkably, an increasing number of studies exploiting these technologies have started revealing the significance of context when it comes to interpreting GPCR signalling effects [10]. In particular, they have highlighted the importance of examining receptor function at a subcellular, temporal, and cell-type specific resolution [11–13].

## Subcellular context and location bias

**Figure 1.**
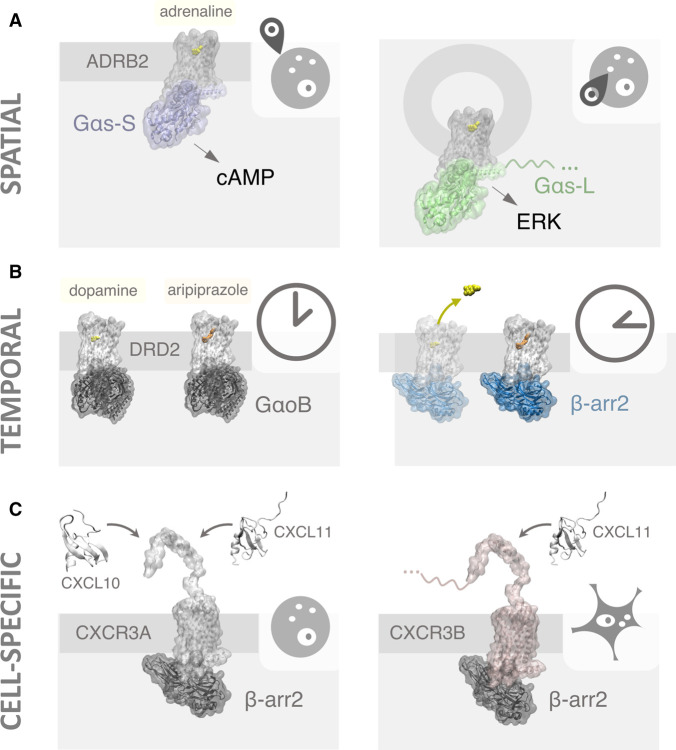
Schematic representation of context-specific GPCR signalling at the spatial, temporal, and cell-specific level. (**A**) Spatial segregation of receptors can lead to compartment-specific coupling and signalling. For instance, recent work has suggested that, apart from the established cAMP signalling of the β2-adrenoceptor (ADRB2) from the plasma membrane, ERK signalling could be associated to endosomal receptor signalling specifically via the long Gαs splice variant (Gαs-L). (**B**) Differences in residence time between endogenous ligands and drugs have been related to observations of temporal bias. This is the case of dopamine and antipsychotic drug aripiprazole at the dopamine D2 receptor (DRD2) and their capacity to promote G protein (GαoB) vs. β-arrestin 2 (β-arr2) coupling. (**C**) Cell or tissue-specific expression of different isoforms of the same receptor can diversify signalling outputs. In the case of chemokine receptor CXCR3, the different capacities of isoforms A and B to respond to endogenous ligands like CXCL10 mean that variation in isoform expression between cell types could lead to cell-specific β-arrestin 2 coupling.

Initial indications of the importance of GPCR function away from the plasma membrane originated from observations of second messenger signalling mediated by internalised receptors [14]. This included evidence on how GPCRs, like the thyroid-stimulating hormone receptor, could be internalised into pre-Golgi compartments in primary cells together with ﻿G protein αs (Gαs) subunits and adenylyl cyclase [15]. Cyclic adenosine monophosphate (cAMP) production patterns and cytoskeleton remodelling could be altered by impairing this internalisation, highlighting the functional role of receptor signalling from intracellular compartments. Subsequent studies with the β2-adrenoceptor revealed that endosome-based cAMP signalling is key to initiate full transcriptional responses downstream of this prototypical GPCR [16]. This led the authors to propose that spatial encoding of receptor signalling can diversify receptor function. In this way, the same ligand could activate different receptor-mediated responses depending on subcellular location giving rise to ‘location-biased signalling’. Interestingly, recent work has described another instance of β2-adrenoceptor location bias, as endosomal signalling from these receptors has been implicated in their capacity to activate extracellular signal-regulated kinase (ERK) [17]. ERK activity seems to be initiated exclusively from endosomes — and not the plasma membrane — and depends on receptor coupling to the long, but not the short, Gαs splice variant (Figure 1A). This observation is particularly relevant for GPCR pathophysiology, as mutations in splicing factors observed in myelodysplastic syndrome selectively increase the expression of long Gαs and drive abnormal signalling through ERK. In this way, dysregulated location bias could be a source of pathological signalling in this condition.

Examples of location bias have also been observed for receptor populations that do not depend on internalisation. In the case of the metabotropic glutamate receptor 5, activation of intracellular receptor pools in rat hippocampal neurons showed location-specific effects on Ca^2+^ signalling and long-term depression [18]. For β1-adrenoceptors, authors observed cAMP signalling originating from the Golgi that was independent of plasma membrane receptor activation or internalisation [19]. These studies also highlighted an aspect of location-specific signalling that is crucial for GPCR pharmacology: if receptors are capable of signalling from intracellular compartments independently from internalisation, their ligands must be able to access such compartments by crossing the plasma membrane. In the case of β1-adrenoceptors, the authors observed that endogenous and exogenous ligands may do so by different mechanisms. While norepinephrine can cross the plasma membrane via the organic cation transporter 3 (OCT3), other exogenous drugs can do so by virtue of being hydrophobic and passively diffusing through the membrane. This leads to the interesting observation that different drugs like β-blockers could access different receptor pools depending on their physicochemical properties. This, in turn, would result in variation in the overall signalling effects of different β-blockers depending on the subcellular locations they are able to access. Such differences in location-dependent signalling could be behind the divergent therapeutic efficacies among β-blockers in the clinic. Furthermore, these observations also point to the possibility of selecting drug candidates according to their capacity to access receptors in different cellular compartments as a way of rationally modulating location-specific GPCR responses [20].

Further studies analysing how receptor signalling from specific locations can contribute to different therapeutic and disease-related phenotypes have exemplified how this idea could be exploited for rational drug design. In the case of β1-adrenoceptors, preventing activation of the receptor in the Golgi by inhibiting norepinephrine entry into the cell through OCT3 could reduce cardiac myocyte hypertrophy in heart failure [21]. For opioid receptors, the endosomal receptor pool could potentially act as a new target for the management of pain [22]. In particular, δ-opioid receptor agonists activating the endosomal receptor pool have been shown to provide a sustained inhibition of nociception in inflammatory conditions [23]. Interestingly, further studies on the δ-opioid receptor using different conformational biosensors have revealed that the receptor can display location-specific conformations upon activation with the same ligand that could lead to compartment-specific receptor coupling [24]. This highlights the importance of investigating ligand mediated GPCR activation in diverse subcellular contexts to fully characterise and exploit therapeutically relevant receptor signalling.

Differential subcellular location of receptors and their ligands, however, is not the only possible source of location bias of GPCR signals. As an example, recent work has elegantly demonstrated how, at low agonist concentrations, second messengers like cAMP can themselves exist in spatially segregated pools [25]. By using a set of molecular nano-rulers [26], researchers have determined how activation of the glucagon-like peptide-1 receptor can give rise to receptor-associated independent cAMP nanodomains (RAINs). Remarkably, signalling from RAINs is strongly influenced by both phosphodiesterase activity and protein kinase A tethering to these nanodomains, highlighting the fact that compartmentalisation can be orchestrated at different points of a GPCR signalling pathway.

## Minding the temporal dimension

The ability to systematically measure receptor function and signalling bias with increasing accuracy has also revealed the importance of time in GPCR activation [27]. A significant example of this originated from a study focused on exploring why both β-arrestin-biased agonism and antagonism had been associated to antipsychotic effects at dopaminergic D2 receptors [28]. To try to explain these seemingly conflicting results, researchers carefully characterised the capacity of an array of ligands to mediate different receptor coupling and signalling outcomes. These included cAMP production, GαoB activation, β-arrestin coupling and changes in cellular impedance at different timepoints. Their results showed how several ligands, including the endogenous agonist, dopamine, displayed unbiased or biased signalling depending on the timepoint at which signalling responses were measured. By comparing the dissociation constants of the different ligands, the authors observed that agonists with slower dissociation kinetics displayed bias towards coupling partners or downstream responses that occurred at longer timescales (Figure 1B). Such differences were attributed to the fact that receptor occupancy for those slow-dissociating ligands would be higher when late coupling or signalling events needed to take place. This led the authors to point out that accounting for ‘kinetic context’ is crucial to systematically characterise receptor signalling in response to biased agonists [29]. In recent years, this realisation has led to increased efforts to incorporate kinetic analyses into the characterisation of GPCR ligands [30].

However, regulation of GPCR spatio-temporal signalling is not only constrained to ligand-dependent effects. In this sense, a high-throughput characterisation of heterotrimeric G protein composition using optical biosensors has revealed how GPCR interaction with G proteins formed of different Gα, β and γ combinations can influence receptor activation kinetics and signalling efficacy from different cellular compartments [31]. In particular, the identity of Gγ in the heterotrimeric complex was shown to determine the speed of translocation to intracellular membrane compartments, the signalling efficacy from different organelles, and the Gα deactivation rates mediated by regulator of G protein signalling proteins. This suggested that preferential expression of specific Gγ subunits could provide cells with a fine-tuning mechanism allowing the spatio-temporal regulation of GPCR responses.

## Cell-type specific signalosomes

Cellular heterogeneity in pathway composition — i.e. the formation of cell-specific ‘signalosomes’ — has been the focus of several studies in the past years. Although GPCRs are generally expressed at low levels [32], advances in sequencing techniques, together with a series of studies specifically designed to monitor receptor expression, have started revealing how coexpression of different pathway components in a tissue or cell-specific manner can greatly impact signalling responses. This was beautifully shown in a study of GPCR single cell expression in murine primary vascular cells [33]. The authors observed how individual cell types displayed high heterogeneity in receptor expression and measured how anatomical location of endothelial and smooth muscle cells determines their GPCR repertoire in both basal and pro-inflammatory conditions. These results highlighted the therapeutic potential of targeting specific cell subpopulations to treat this and other pathological phenotypes.

Another study focused on monitoring single cell heterogeneity in the GPCR system focused on analysing the expression of genomically encoded neuropeptidergic receptor ligands and their GPCRs in mouse neocortical neurons [34]. ScRNAseq revealed the neuron-type-specific expression of 37 cognate pairs amongst 18 neuropeptide precursors and 29 neuropeptide-selective GPCR genes. These pairs would constitute a directed modulatory network with nodes defined by the neurotaxonomic identities of the neuron producing the neuropeptide and the neuron expressing its corresponding receptor. Notably, detailed cell type-specific ligand–receptor networks like the ones presented in the study could help optimise further targeted experiments focused on analysing local signalling responses in highly specific organ and tissue sites.

Systematically monitoring receptor expression has also revealed that most cell types contain tens of GPCRs at a time, opening the intriguing question on how the joint expression of these different receptors impacts cell signalling. This could be particularly critical in the case of scavenger receptors like ACKR3/CXCR7 [35]. This receptor was previously believed to act as a chemokine scavenger but has now also been described as a receptor for a series of opioid peptides. In this way, coexpression of opioid receptors and ACKR3 in different brain regions could dampen the signals of endogenous opioids like enkephalins and dynorphins, as some of these signals would be sequestered by ACKR3. The authors also used a selective agonist towards ACKR3 to increase availability and signalling of opioid peptides, providing proof-of-concept that targeting this scavenger receptor could represent a therapeutic strategy to increase opioid receptor function. Further work from the same group has more recently suggested chemokines and opioid peptides may not be the only GPCR ligands capable of binding and activating ACKR3. Specifically, class B receptor ligands like adrenomedullin and proadrenomedullin N-terminal 20 peptide (PAMP) seem to also bind to, and in the case of PAMP, also activate ACKR3 [36]. All these findings point to the importance of assessing the combined expression of GPCRs and their potential ligand scavengers to understand signalling responses in specific cell types.

A more subtle example of how combinatorial receptor expression can alter ligand responses comes from our recent work on GPCR isoforms [37]. Many receptors exist as a series of structurally and functionally distinct isoforms that show differential ligand binding, efficacy, coupling and trafficking properties. As an example, different isoforms of the chemokine receptor CXCR3 have been found to differentially couple to β-arrestin 2 [38]. While both CXCR3A and CXCR3B isoforms display this coupling in response to CXCL11, only CXCR3A can respond to CXCL10 (Figure 1C). By systematically analysing receptor isoform expression across 30 different tissues from hundreds of human donors, we observed that receptors mostly varied in regions related to ligand binding and receptor interaction with transducers. This, combined with the fact that not all receptor isoforms were found to be consistently expressed across tissues, points to combinatorial receptor expression as a source of signalling variability. In particular, activation of different isoform pools with distinct structural and functional properties by the same compound in a series of tissues, could mean that individual natural ligands or drugs could yield differential context-specific signalling responses. On the other hand, locating highly cell or tissue-specific receptor isoforms with differential ligand binding interfaces, could provide an exquisite source of site specificity for new GPCR drug candidates that could avoid on-target side effects in disease-unrelated tissues.

Characterising cell-specific differences in receptor composition, however, may not always be sufficient to predict downstream effects. Using zebrafish primordial germ cells to measure a series of phenotypic readouts related to embryonic development, a recent study suggested that single chemokine receptors may be able to activate a number of responses in different cell types, while specific phenotypes may be activated by multiple types of chemokine receptor when expressed in a given cell [39]. The authors justified these unexpected observations by proposing that different cell types can contain specific chemokine receptor signal interpretation modules that can be activated by generic signals produced by multiple chemokine receptors. The existence of such signal interpretation modules would underscore the importance of reconstructing full cell specific GPCR activation pathways in order to relate receptor activation to molecular phenotypes.

## Signalling response in a changing environment

Thorough investigations on cell-type specific GPCR responses over space and time have revealed crucial details about receptor function under different physiological and disease-related conditions. In some cases, context can even regulate receptor signalling in the absence of an endogenous ligand. In the gut microenvironment, for instance, receptor ligands can be generated via molecular mimicry by our bacterial flora. As an example, work focused on the production of N-acyl amides by the human gut microbiota detected compounds like N-acyl serinols, which are capable of binding GPR119 to regulate blood glucose levels and glucagon-like peptide-1 secretion in mouse models [40]. Another noteworthy study on receptor modulation by physiological context includes recent work on how cold temperature itself could be the key regulator of GPR3 function [41]. In their study, researchers determined how a reduction in temperature led to increased lipolytic activity in brown adipose tissue resulting in increased expression levels of GPR3. This receptor, in turn, could be constitutively activated by its own N-terminus and signal via Gs proteins to promote thermogenesis, having a protective effect against metabolic disease.

Another intriguing example of receptor activation in response to environmental change comes from GPCRs capable of acting as mechanosensors. This includes receptors that can be activated by shear stress in the absence of their endogenous ligands like class A angiotensin II type 1 and sphingosine-1-phosphate receptors, as well as adhesion receptors such as ADGRE5 and ADGRG1 [42]. Recent work focused on the role of the histamine H1 receptor (H1R) as an endothelial mechanosensor has delved into which structural receptor features allow mechanosensitivity [43]. The authors describe how the receptor helix 8 appears to be crucial for the detection of shear stress, with the deletion of the helix rendering receptors irresponsive. Furthermore, the inclusion of the helix 8 of H1R into a receptor chimera of the insensitive human gonadotropin-releasing hormone receptor (GnRHR) resulted in a gain of mechanosensory properties. This is particularly intriguing considering that other organisms, like pigs, can express two distinct GnRHR isoforms that either include or exclude helix 8, thus changing their ability to act as mechanosensors. In more general terms, all these observations open thought-provoking questions on how endogenous ligand and mechanical signals can be jointly integrated by a number of receptors to produce highly context-dependent signalling patterns.

Importantly, pathological microenvironments can also be the source of context-dependent receptor activation. In a recent study, the mouse olfactory receptor Olfr2, and its human homologue OR6A2, together with their downstream signalling machinery, were found to be expressed in vascular macrophages from atherosclerotic arteries [44]. This receptor signals in response to octanal, which can be generated by lipid peroxidation in the aorta. Receptor activation by octanal in vascular macrophages led to inflammatory responses, whereas Olfr2^−/−^ mice displayed reduced plaque formation. These results pointed to OR6A2 as a potential target to treat, prevent or reverse atherosclerosis.

Therapeutic exploitation of receptor microenvironments themselves has also been suggested as a potential source of drug selectivity. This is the case for acidified microenvironments that are generated during inflammatory processes. In a recent study, researchers analysed a previously developed fentanyl analogue, N-(3-fluoro-1-phenethylpiperidine-4-yl)-N-phenyl propionamide (NFEPP), which had been rationally designed to change protonation state between pH 6.5 and 7.4, so that it would activate peripheral μ-opioid receptors in injured acidified tissues, while sparing the receptor in healthy tissues displaying physiological pH levels [45]. By characterising NFEPP in a mouse model of inflammatory bowel disease, the authors could observe how this compound preferentially activated µ-opioid receptors in the acidified microenvironment of inflamed tissues, thus causing antinociceptive effects, but didn't cause other symptoms generally associated with fentanyl like respiratory depression, constipation and hyperactivity [46]. These results point to the importance of characterising GPCR signalling in relevant disease scenarios to fully understand and exploit context-specific therapeutic opportunities.

## Conclusions

Taken together, the increasing number of observations on context-dependent GPCR signalling is shedding new light into key unresolved questions in the receptor signalling field: how can activation of a single receptor by its endogenous ligand give rise to such a diverse array of cellular responses? And how is the selective activation of some of these responses encoded and regulated in a cell? At least partly, the answer to those questions can be related to key differences in the spatial, temporal and cell-specific environment in which that ligand–receptor interaction occurs. Although these context-dependent effects can appear separately in individual receptor types, they most often combine to give rise to highly specific signalling outputs.

Incorporating these considerations into the study of receptor function can be expedited by new experimental advances in the GPCR field allowing to monitor how receptor-partner interactions evolve over time though techniques like GPCR–APEX [47], to closely investigate the dynamics of these interactions via single-molecule imaging [48], or to precisely time receptor activation patterns via photopharmacology [49,50]. Furthermore, ongoing efforts to reconstruct cell-specific GPCR signalling pathways [51] can also shed light into how changes in the composition of the signalosome diversify receptor outputs. Crucially, we cannot expect synthetic ligands, including drugs in the clinic, to behave like GPCR endogenous ligands when it comes to this subtle context-dependent signalling regulation. We can, however, try to dissect context-specific signalling processes and their phenotypic relevance to exploit them as a source of selectivity in future GPCR drug candidates.

## Perspectives

- Variability in GPCR responses found in distinct spatial, temporal and cell-specific contexts is increasingly emerging as a physiological mechanism for the diversification of receptor signals in response to endogenous ligands.
- Employing experimental and theoretical approaches that account for this GPCR context-dependent signalling can reveal key functional differences between natural and synthetic receptor modulators, as well as among different drugs or drug candidates targeting a specific receptor.
- A more nuanced view linking receptor signalling in context to particular molecular phenotypes holds new opportunities for the selection of new and more selective GPCR drug candidates with rationally constrained mechanisms of action.

## Open Access

Open access for this article was enabled by the participation of University of Cambridge in an all-inclusive *Read & Publish* agreement with Portland Press and the Biochemical Society under a transformative agreement with JISC.

## Abbreviations

cAMP: cyclic adenosine monophosphate
ERK: extracellular-signal-regulated kinase
GPCR: G protein-coupled receptor
Gαs: G protein αs
NFEPP: N-(3-fluoro-1-phenethylpiperidine-4-yl)-N-phenyl propionamide
OCT3: organic cation transporter 3
PAMP: proadrenomedullin N-terminal 20 peptide
RAINs: receptor-associated independent cAMP nanodomains
